# Emergency Preparedness and Response in Occupational Setting: A Position Statement

**DOI:** 10.3389/fpubh.2017.00251

**Published:** 2017-09-21

**Authors:** Alexis Descatha, Susanne Schunder-Tatzber, Jefferey Burgess, Pascal Cassan, Tatsuhiko Kubo, Sylvie Rotthier, Koji Wada, Michel Baer, EPROH Scientific Committee, Nicholas AIGBOVO

**Affiliations:** ^1^AP-HP, EMS (Samu92), Occupational Health Unit, Raymond Poincaré University Hospital, Garches, France; ^2^University of Versailles Saint-Quentin-en-Yvelines, Versailles, France; ^3^INSERM, UMS 011 UMR1168, Villejuif, France; ^4^Corporate Health Management, OMV AG, Vienna, Austria; ^5^Austrian Academy for Occupational Health & Prevention, Klostenreuburg, Austria; ^6^Mel and Enid Zuckerman College of Public Health, University of Arizona, Tucson, AZ, United States; ^7^Global First Aid Center, International Federation of Red Cross and Red Crescent Societies, Paris, France; ^8^Department of Public Health, University of Occupational and Environmental Health, Kitakyushu, Japan; ^9^La Poste Service Medical/Groupement Infirmier du Travail (GIT), Paris, France; ^10^Bureau of International Health Cooperation NCGM, Tokyo, Japan

**Keywords:** disaster, prevention, training, emergencies, occupational health

## Background and Rational

Accidents and acute medical events at workplaces are unfortunately not rare events—the International Labour Organization (ILO) estimates that 313 million people are injured and 350.000 are killed in work-related accidents annually (1). The World Health Organization (WHO) has called for action dealing with all aspects of workers’ health, including primary prevention of occupational hazards, protection and promotion of health at work, improved employment conditions, and a better response from health systems to workers’ health (2).

The Emergency Preparedness and Response in Occupational Health (EPROH) scientific committee was developed as part of the International Commission on Occupational Health (ICOH) (3) to raise awareness of emergency risks for workers, and to train managers, employees, and medical staff to prevent accidents, and to mitigate their impact.

Its work is guided by the following principles:

– Every worker worldwide should have access to first aid, rescue, and emergency care in case of accidents and acute medical events.
– Compliant with “ICOH policy”–In the “Seoul Statement (June 2015) on the development of Occupational Health and Safety for All”–and approved by ICOH, it is stated that Occupational Health and Safety includes prevention of physical and mental diseases and injuries inclusive of first aid (4).
– Influenced by the model of the chain of survival, where response and care are linked to preparedness, multidisciplinary cooperation between occupational/safety professionals, on the one side, and emergency responders/volunteers, on the other side, will harmonize first aid and emergency care approaches worldwide (5).

The EPROH committee follows Evidence Based Medicine principles and undertakes literature review, practice networking, and surveys, including workers’ expectations to develop recommendation for prevention, first aid treatment, and additional care within the field of occupational health. All potential conflicts of interest are mandatorily stated and reviewed.

To review the existing knowledge about emergency response, we started to look for the best evidence from research and performed systematic reviews on some aspects of regulation, procedures, and training in first aid, including effectiveness of automated external defibrillators in workplaces (6, 7). Considering the publication bias taking into account few countries that published and evaluate their own procedures (8–12), we have sought for sources of clinical expertise and experience, including field actors (13, 14). For these purposes, the EPROH committee aligns with other scientific bodies and organizations and performed survey on representative members to have more information of diversity of practice and procedure in that field. Though surveys design limited the interpretation, the very few existing procedures, specific training, prevention of emergencies in occupational health all around the world, encouraged us to draw this position statement.

## Scope of Position Statement

Taking into account the title of our group “Emergency Preparedness and Response in Occupational Health,” we clarified the scope of our group. We aim to be involved in practice, training, and research on:

- Emergency Plans and Procedures
  These plans and procedures range from daily care to exceptional emergency care situations. They have to respond to risks by the way of an appropriate assessment, taking into account local legislation, cultural and organizational specific policies, economic background, number of employees, contractors, and local emergency medical services (EMS) systems. These plans and procedures must include:
  ○ Medical supplies and equipment—such as first aid kits, automatic external defibrillators (AEDs), drugs for emergency care, respirators, portable medical equipment, tele-medical equipment, appropriate personal protective equipment (PPE) for medical staff, ambulances, and additional vehicles as needed.
  ○ Infrastructure—e.g., field clinics, first aid stations, and muster points.
  ○ Human resources—minimal number of first aiders, paramedics, nurses, and doctors as well as distribution of work shifts.
  ○ Organizational plans—including alarm plans, plans of installations, means of communication, plans for maintenance of medical equipment and replacement of medical goods, and development of “emergency medical guidelines” on medical care and “fact sheets” on chemicals employed.
  ○ Cooperation with other internal/external rescue and public/private services (e.g., fire departments), and EMS systems incl. medevac procedures. In case of disaster, plan to accept external assistance of occupational health services, including human resources.
  ○ Provision for mass casualties and disaster care.
- Preparedness and Training
  ○ Occupational health provisions for emergency care providers—such as immunizations, training aimed at site-specific risks, PPE, and access to specific health promotion programs.
  ○ Training plans to build and maintain skills and competencies—including first aid courses, training for medical staff, medical emergency drills, and cooperation with internal and external rescue teams and other care providers.
  ○ Audit procedures—including plans for internal and external audits to ensure quality and continuous improvement.
  ○ Communication lines—internal and external, including media info and contacts.
  ○ Refresher courses and compulsory « long life learning » during the working time.
- Prevention of emergencies and follow-up
  ○ Risk assessment: medical emergency care needs at workplaces should be identified through a thorough risk assessment. This includes physical, chemical, ergonomic, work organization, and environmental influence factors, such as climate and epidemic problems. This assessment must take into account local emergency response characteristics such as time for EMS to reach the scene and time to reach medical facilities by any means of transportation, such as by road, sea, air, and so on.
  ○ Surveillance system based on risk assessment and adverse health effects to trigger an alert response in case of a potential emergency occurred.
  ○ Psychosocial care—debriefing preparedness sessions should address both involved employees and medical staff if needed. This will provide opportunities to gain access to trained psychological resources in case of unexpected need.
  ○ Special follow-up of responders as needed.
  ○ Use of reporting tools and lessons learned.

**Figure 1:**
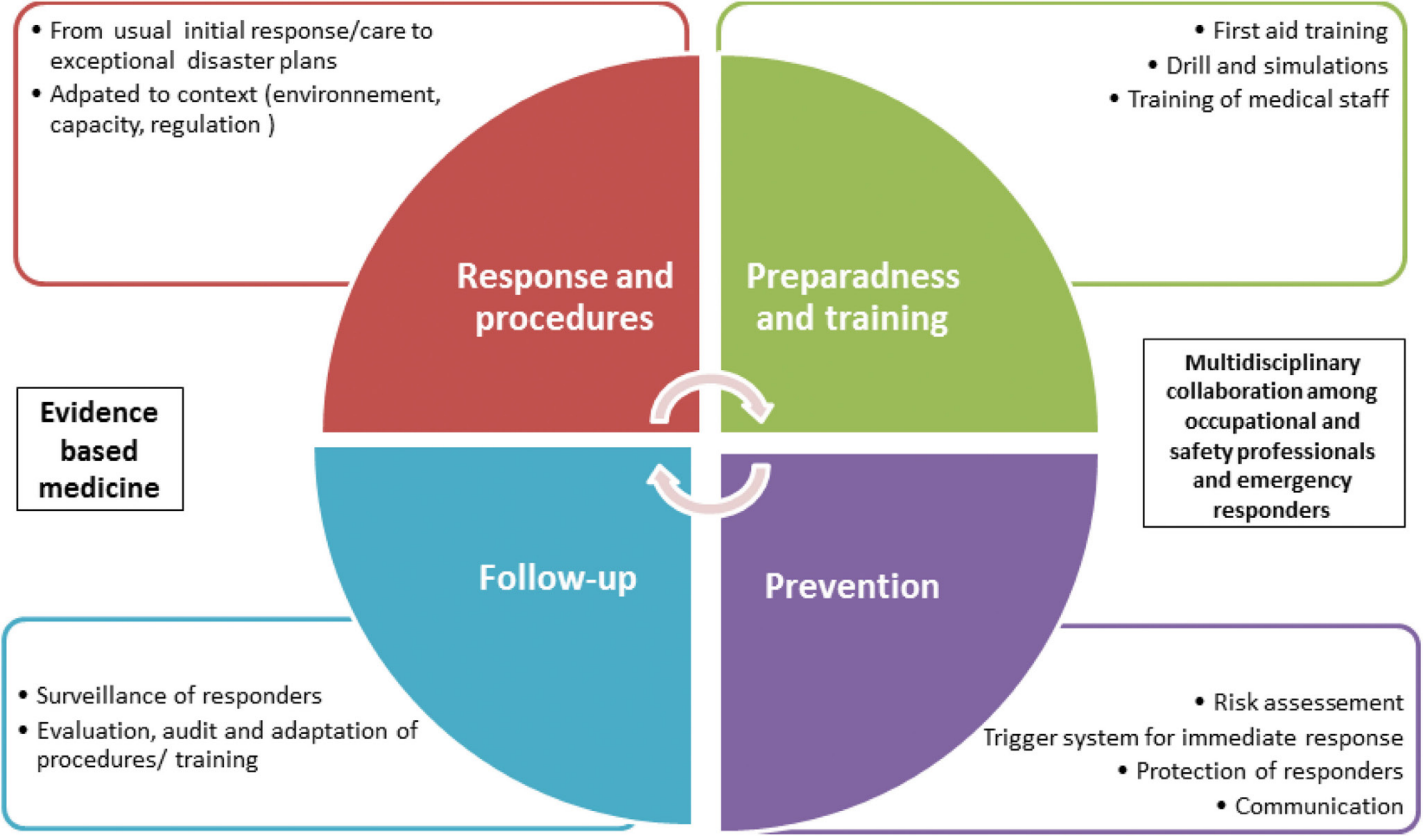
Scope of the statement.

All of these elements are linked together for the purpose of improving workers’ health and safety from those injured to responders (Figure 1). We will reach these goals by optimizing emergency response with appropriate procedures, by reinforcing preparedness with training and first aid implementation, and by increasing prevention and follow-up of workers and responders, before and after emergencies.

## Statements

To improve the dramatic situation of workplace fatalities and accidents/events, EPROH experts have developed the following general position statement:

- Each worker should benefit in case of a medical emergency, work related or not, minor or major, from an optimal response delivered by the company until emergency medical services (EMS) arrive on scene.
- Minimum response plans for every workplace include information about initial management and EMS contact information. The duty of the employer is to deliver initial care until EMS arrives to handle the case.
- Information about initial care in case of emergency must also include a procedure for contacting other rescue services when EMS is not available such as fire departments, law enforcement, or other first responders.
- First aid must be encouraged, and several steps may be identified:
  ○ Minimum traceable information made available to all workers with description of the company’s procedures in case of emergency.
  ○ Basic Life Support responses based on International First Aid/International Liaison Committee on Resuscitation (ILCOR) recommendations, with modules adapted for specific company risks.
  ○ Refresher courses during the working time.
- Advanced Life Support when available, either incorporated in the company or sub-contracted to other bodies.
- Occupational health and safety professionals will have to develop procedures that detail responses to emergencies, from minor events to major disasters, including alert systems. The written procedures should be based on initial symptoms and adapted to the company-specific situation and shared between professionals and signed by them, and reevaluated regularly.
- As needed, emergency providers should be followed up regularly by an occupational specialist for extended intervals after an event, on a regular basis and sometimes for a prolonged period of time. In addition to fitness for work, specific training should focus on information about chemical, physical, biological, and psychological hazards and risks. Regular simulation sessions and drills should be a mandatory component of the training.
- In case of mass casualties/disaster incidents, reasonable but rapid communication aimed at professionals and volunteers, on the one side, and media, on the other side, must be released by involved companies, assisted by occupational professionals.
- Public policies should help companies to implement such programs of prevention and response to emergencies in occupational settings. Financial discounts of insurance, special offers for best prevention, or other incentive programs should be encouraged.
- Evaluation should be performed at least every 5 year.
- We are emphasizing the importance of business continuity plan. OH can support for making this plan.

## Discussion

This position statement emphasizes the importance of having a general position statement on the multiple aspects of emergency preparedness and response in occupational settings for every worker.

Previous surveys and reports highlighted the difference of general organization of Health and Work systems, mostly on the important question related on “who are in charge in Emergency Preparedness and Response in Occupational Health”: is it companies itself, the government or community, or nobodies. Then, this general position statement should be adapted locally with the help of national societies in charge of emergency response and occupational health and safety. Subcontracting companies involved in first aid, rescue and care in occupational setting should comply with these requirements and adapt their own procedures to avoid discrepancies.

Additional research and global harmonization is needed for emergency preparedness and response in occupational settings. The current level of evidence is limited, mostly based on practice experience and workers expectations, with few global recommendations. The difference of regulation in nations, in workplaces—such as from the size of the company, its activity, and financial capacity, environment, and emergency response capacity, makes harmonization challenging.

However, this general position statement should be applied in every country with the help of national societies in charge of emergency response in occupational settings or other relevant not-for-profit organizations. It is a first step in the long road of improving general worker health with the help of everyone.

## EPROH Scientific Committee

Emergency Preparedness and Response in Occupational Health (EPROH) scientific committee: Nicholas Aigbovo; Fathi Ben Larbi; Nick Copper; Maurits De Ridder; Dorothy Dingwiza; Félix Enrique Echevarria Reymer; Ahmed El Makaty; Babacar Fall; Wahida Farah; Mame Diarra Faye; Diana Gagliardi; Todd Hamel; Philippe Havette; Richard Heron; Mouloud Kalaai; Margaret Kitt; Tatsuhiko Kubo; Roberto Lucchini; Ashish Mittal; Horatiu Moldovan; Akiba Okon; Anna Ozguler; Bruno Papaleo; Kumar Selva; Promila Sharma; Mukhtar Syed Aamir; Styliani Tziaferi; Paula Viapiana; Koji Wada; Joy Wilson; Barthelemy Wognin.

## Author Contributions

All authors had substantial contributions to the conception and interpretation of the work. Investigator list (EPROH scientific committee) includes all active participants of the group. All authors gave final approval of the version submitted.

## Conflict of Interest Statement

No conflict of interest (authors are paid by their affiliations, and involved in training, research and practice in the Emergency or occupational field. AD is also paid to be Editor in chief of “les archives des maladies professionnelles et de l’environnement”).
